# Pulp transplantation in necrotic mature teeth with periapical radiolucency

**DOI:** 10.1007/s00784-026-06850-7

**Published:** 2026-03-28

**Authors:** Elife Ülkü Tatar, Esra Balkanlioglu, Aliye Kamalak

**Affiliations:** Department of Endodontics, Kahramanmaras Sutcu Imam University, Avsar Campus No: 251, A Onikisubat, Kahramanmaras, Turkey

**Keywords:** Concentrated growth factor (CGF), Mature necrotic teeth, Autologous pulp transplantation, Dental pulp transplantation, Regenerative endodontics

## Abstract

**Objectives:**

This exploratory randomized clinical trial evaluated the regenerative potential of autologous dental pulp transplantation, with or without concentrated growth factor (CGF), compared with conventional root canal therapy (RCT). The aim was to assess sensibility recovery and periapical healing in necrotic mature teeth with periapical radiolucency.

**Materials and methods:**

Twenty-one patients were randomly allocated into three groups (*n* = 7): autologous pulp transplantation, pulp transplantation with CGF, and conventional RCT. Donor pulp tissues were obtained from non-carious third molars. Follow-up examinations were performed at 3, 6, and 12 months. Sensibility was assessed using electrical pulp testing, and periapical healing was evaluated by periapical radiographs and cone-beam computed tomography. Statistical analyses included non-parametric tests (Kruskal–Wallis and Wilcoxon) and categorical comparisons (chi-square/Fisher’s exact) (α = 0.05).

**Results:**

Positive sensibility response was observed in 71.4% of teeth in the pulp transplantation group and 42.9% in the CGF group, while no sensibility recovery occurred in the RCT group. All groups showed periapical healing, with the greatest lesion reduction in the pulp transplantation group (*p* < 0.01). No adverse events were reported.

**Conclusions:**

Autologous dental pulp transplantation appears to be a safe and promising approach for necrotic mature teeth, whereas the addition of CGF did not provide additional clinical benefit in this cohort.

**Clinical relevance:**

Autologous dental pulp transplantation may represent a viable regenerative alternative to conventional RCT in necrotic mature teeth, with the potential to promote periapical healing and partial sensibility recovery without additional biological adjuncts.

## Introduction

Regenerative endodontics represents a modern therapeutic approach that seeks to restore tooth vitality by excising necrotic or diseased pulp tissue and substituting it with healthy, functional pulp tissue [1]. This biologically driven methodology is based on the integration of three essential components of tissue engineering: stem cells, biocompatible scaffolds, and biomolecular signals. Advances in mesenchymal stem cells (MSCs)–based technologies have further accelerated progress in this field [2]. Regenerative endodontic procedures (REPs) were originally developed to support root development in immature teeth. These procedures have shown favorable clinical and radiographic outcomes and are now being investigated for use in mature teeth [3].

Recent studies have demonstrated that regenerative therapies can be effectively applied to mature necrotic teeth, where the stage of root development does not constrain clinical success, and both clinical and radiographic healing have been observed [4, 5]. Interest in applying regenerative techniques to mature teeth has increased due to several advantages, including reduced iatrogenic complications, decreased heat generation during canal preparation—particularly in curved canals—and the potential to shorten clinical procedures [6]. Case series have observed the resolution of apical periodontitis, the restoration of periapical tissues, and favorable sensibility responses subsequent to the regenerative treatment of mature teeth [7]. However, exploratory randomized clinical evidence in this population remains limited, with most available data derived from small case series and non-comparative studies.

Tooth-derived stem cells, such as dental pulp stem cells (DPSCs), stem cells from human exfoliated deciduous teeth (SHED), stem cells from the apical papilla (SCAP), and periodontal ligament stem cells, represent some of the most promising sources of cells currently available. This is attributed to their significant proliferative capacity, multipotency, accessibility, and the lack of ethical concerns [8, 9]. DPSCs can be isolated from routinely extracted teeth, such as third molars or deciduous teeth [10, 11]. These teeth are often considered medical waste, and the cells may retain viability even after cryopreservation. These characteristics make cell-based regenerative therapies highly attractive for clinical translation. The technique implemented in this study is consistent with evidence showing that the pulp tissue serves as a rich reservoir of MSCs capable of de novo pulp–dentin regeneration without the need for in vitro expansion [12].

Beyond stem cell–based therapies, several biomaterials and biomolecules—including platelet-derived products such as platelet-rich fibrin (PRF), platelet-rich plasma (PRP), and concentrated growth factor (CGF)—are being explored to enhance the regenerative process [12, 13]. CGF, an autologous concentrate rich in growth factors. It has been reported to support cell proliferation and migration, facilitate the resolution of apical periodontitis, promote pulp–dentin complex repair, and enhance neurovascularization [14–16]. Nevertheless, most available studies are characterized by relatively small cohorts and insufficient follow-up periods, preventing a comprehensive assessment of long-term clinical outcomes [17, 18]. Notably, the adjunctive use of CGF in combination with autologous pulp transplantation has not yet been evaluated in a clinical randomized setting.

Although current evidence suggests that regenerative endodontics can be applied to mature necrotic teeth, treatment success depends on the choice of cell source and biomaterials. Clinical evidence regarding pulp transplantation in mature permanent teeth remains extremely limited, and no randomized study has directly compared pulp transplantation alone with pulp transplantation supplemented by CGF. This research sought to investigate the regenerative capacity of dental pulp transplantation in the revitalization of necrotic mature teeth with periapical radiolucency, to assess the role of CGF in this process, and to compare the results with those obtained from traditional RCT.

## Materials and methods

### Patient selection and ethical approval

This exploratory randomized clinical trial included 21 patients who presented to the university dental hospital with periapical radiolucency in their single-rooted teeth. All individuals participating in the study had caries-free and periodontally healthy third molars that could be used as donor pulp sources. The Institutional Ethics Committee granted approval for the research protocol (Decision No. 11/ 2023). Clinical trial was registered under the ID NCT07258888. Before participating, all individuals received a comprehensive briefing on the study’s objectives, the available treatment options, and the potential risks, following which written informed consent was obtained. This study was conducted in accordance with the principles set forth in the Declaration of Helsinki and its subsequent amendments. This study followed a fixed protocol, and no modifications were made after enrollment. To ensure consistency and standardization in the clinical procedures, they were all performed by a single endodontist with over ten years of clinical experience at the Department of Endodontics, Faculty of Dentistry, XXX University.

### Sample size calculation

In the absence of robust prior randomized data specific to pulp transplantation in mature necrotic teeth, reliable assumptions for effect size and variance were not available to support a conventional a priori power calculation. Therefore, the present study was designed as an exploratory randomized clinical trial, and the sample size was determined pragmatically based on feasibility and ethical considerations [19]. This approach is consistent with previous regenerative endodontic studies conducted in mature necrotic teeth that reported clinically meaningful outcomes with relatively small sample sizes [20, 21].

A total of 21 participants (7 per group) was considered appropriate to enable clinical and radiographic evaluation. Effect sizes with confidence intervals are reported to inform future confirmatory trials.

### Randomization and group allocation

Participants were systematically assigned to three distinct groups (*n* = 7 per group) utilizing a computer-generated random number sequence. Due to the distinctly different clinical procedures, blinding of operators and patients was not feasible; however, outcome assessors and statisticians remained blinded. The outcome assessor who evaluated the radiographic images and performed sensibility testing was not involved in the treatment procedures and was unaware of the group allocation. Radiographic images were coded and anonymized prior to evaluation to prevent identification of the treatment group. This sequence was developed by an independent statistician who had no involvement in the clinical or analytical stages of the study. A straightforward randomization technique with an allocation ratio of 1:1:1 was implemented to ensure an equitable distribution of participants across the three study arms: PT group (pulp transplantation), PT + CGF group (pulp transplantation with concentrated growth factor), and RCT group (conventional root canal therapy). To ensure allocation concealment and reduce selection bias, randomization codes were placed in sequentially numbered, opaque, sealed envelopes, which were prepared by an independent research assistant. These envelopes were opened sequentially by the clinician only after the patient had been enrolled and baseline assessments were completed. The personnel responsible for enrolling participants and assigning them to interventions did not have access to the allocation sequence prior to assignment.

The inclusion criteria were as follows: individuals aged 18–40 years without systemic disease and with single-rooted teeth diagnosed with pulp necrosis and associated radiographic periapical lesions. Pulp necrosis was diagnosed based on the absence of response to electric pulp testing and cold testing, together with the presence of clinical and radiographic findings consistent with pulp necrosis, including periapical radiolucency and lack of bleeding upon access cavity preparation. The exclusion criteria were diseases that may affect systemic healing, absence of suitable donor third molars, and presence of advanced periodontal loss.

Donor pulp tissue was obtained from caries-free third molars of patients who required tooth extraction. After clinical examination of the patients, panoramic radiographs were taken of all patients to evaluate both the tooth to be transplanted and the third molar tooth to be used as the donor. The tooth receiving the pulp transplant and the size of the periapical lesions were assessed using periapical radiography and CBCT.

### Clinical protocol

#### First appointment

Local anesthesia (Articaine hydrochloride 40 mg/ml + epinephrine bitartrate 0.009095 mg/ml; Vem Pharma, Istanbul, Turkey) was administered. Isolation was achieved using a rubber dam, and the access cavity was opened. Canal length was identified using an electronic apex locator (Woodpex-3 Gold Plus; Woodpecker, Guilin, China) and radiographically validated. Canal shaping was accomplished using stainless steel K-files ranging from #45 to #80 (Bahadır Dental, Istanbul, Turkey) following a step-back approach initiated coronally and extended apically. Thus, sufficient width was achieved for the placement of the donor pulp tissue into the canal of the tooth.

Irrigation was standardized as follows: 2.5% sodium hypochlorite solution (NaOCl) (Microvem, Altun Medical, Sakarya, Turkey), total volume 10 mL per canal, delivered in 2 mL increments; sonic activation for 30 s after each 2 mL NaOCl application (EndoActivator, Dentsply-Sirona, Bensheim, Germany ).

This concentration was selected in accordance with AAE/ESE recommendations for regenerative procedures, balancing antimicrobial effect with preservation of dentin-derived growth factors.

The canals were dried and medicated with triple antibiotic paste (TAP). The TAP formulation used (ciprofloxacin–metronidazole–cefuroxime) intentionally excluded minocycline to avoid crown discoloration. The cavity was then sealed with a temporary glass ionomer cement (GC Fuji IX; GC Corp, Tokyo, Japan).

#### Second appointment

The patients were recalled three weeks later. An additional disinfection protocol was applied in cases where the infection persisted. In teeth where signs of infection had improved and there were no symptoms, the temporary filling was removed, and the canal was cleaned by irrigation with 2.5% NaOCl (5 mL) and 17% ethylenediaminetetraacetic acid (EDTA) (5 mL). The irrigant was activated using a sonic device (30 seconds per irrigant). The canal was thoroughly dried using sterile paper points. A #25 K-file was advanced 2 mm beyond the apical foramen to induce controlled bleeding, not to create a scaffold, but to provide the essential oxygenation and nutrient supply required for the survival of the transplanted pulp stem cells.

### Donor pulp harvesting and transplantation

The donor third molar was atraumatically extracted under local anesthesia. To remove the pulp without traumatizing it, a longitudinal notch was made on the tooth under copious saline irrigation with a low-speed diamond bur. The tooth was split into two using a sterile elevator, and the intact pulp tissue was removed.

The harvested pulp was gently lifted with microforceps, avoiding traction on the apical vascular bundle, and immediately transferred into the prepared recipient canal to minimize dehydration and mechanical trauma. The pulp was inserted slowly and passively, allowing it to adapt along the full length of the canal without compression. No packing force was applied, and the tissue was advanced coronally only until mild resistance was encountered to prevent disruption of the apical portion. The progression of the pulp tissue within the canal was guided using sterile microforceps and confirmed visually under magnification to ensure that the tissue extended along the full working length of the canal.

A layer of sterile, hemostatic, absorbable gelatin sponge Spongustan(Ferrosan Medical Devices, Soborg, Danimarka) made of lyophilized hydrolyzed collagen was placed on the pulp tissue to serve as a coronal support matrix and prevent migration during MTA placement.

On top of this, a 2–3 mm thick coronal plug was created using mineral trioxide aggregate (MTA) (ProRoot MTA, Dentsply, Johnson City, TN, USA). Mineral trioxide aggregate (MTA) was selected because of its well-documented biocompatibility, excellent sealing ability, and bioactive properties that promote hard tissue formation and periapical healing. MTA has been widely used in regenerative endodontic procedures as a coronal barrier due to its capacity to provide an effective seal while supporting a favorable biological environment for tissue repair and regeneration. These properties make MTA a suitable material for use as a coronal plug in regenerative endodontic approaches [22].

**Fig. 1:**
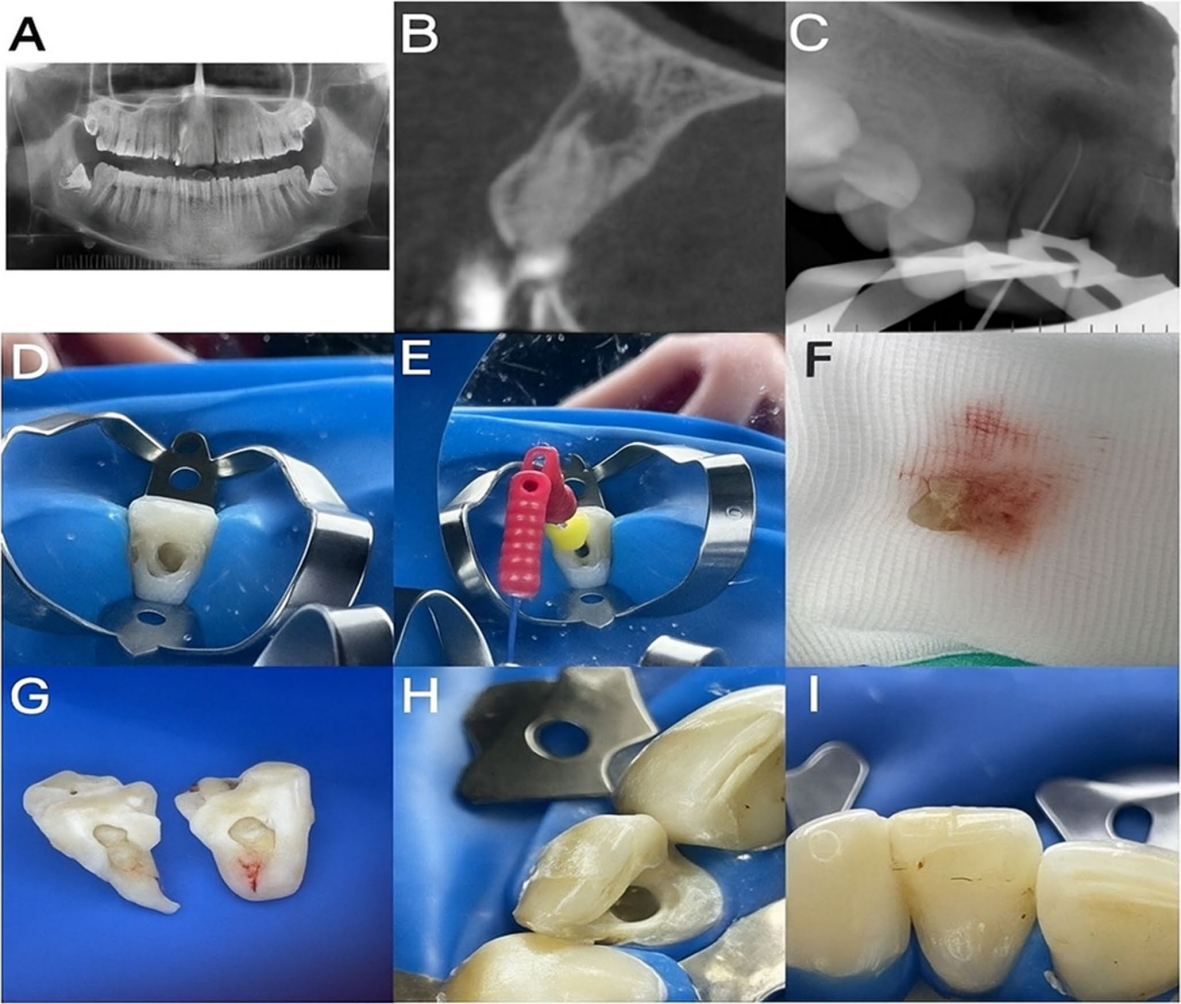
The tooth to be transplanted refers to the third molar, which serves as the donor. The initial periapical and CBCT images of the patient are shown. In the image below: **a**) Preoperative intraoral photograph of the relevant tooth **b**) Photograph after isolation and access cavity preparation in the first session **c**) Determination and shaping of the canal length of the recipient tooth **d**) Obtaining apical bleeding immediately before transplantation in the second session **e**) The patient’s extracted third molar **f**) Separation of the patient’s extracted third molar and exposure of the pulp tissue g)Placement of the pulp tissue into the canal of the recipient tooth, followed by securing the coronal seal with MTA **h**) Restoration of the tooth with composite resin **i**) Immediate postoperative periapical radiograph of the transplanted tooth. The transplanted pulp tissue was gently advanced into the recipient canal using sterile microforceps until resistance was encountered at the working length, ensuring that the tissue extended along the full length of the prepared canal without compression

To allow the MTA to set, a moist cotton pellet was placed for 15 min. The cavity was restored using glass ionomer cement (Fuji II LC; GC, Tokyo, Japan) as a liner, followed by composite resin build-up (Universal Restorative 200; 3 M ESPE, St. Paul, MN, USA). (Fig. 1).

### Preparation of CGF (Group 2)

Venous blood samples were collected from the same patient. The blood samples were centrifuged in a MediFuge device (Silfradent, Santa Sofia, Italy) using the standardized CGF protocol described by Yu et al. [23]. The program consisted of: 30 s of acceleration, 2 min at 2700 rpm (600 g), 4 min at 2400 rpm (400 g), 4 min at 2700 rpm (600 g), 3 min at 3000 rpm, followed by 36 s of deceleration.

Another empty tube containing the same mass of water was used as a balancing tube [17]. Three layers were obtained: platelet-poor plasma on top, a fibrin gel rich in platelets and growth factors (CGF) in the middle, and an erythrocyte layer at the bottom of the tube. The gel obtained from the middle layer was shaped on sterile glass and placed over the pulp tissue. The CGF layer was gently separated using sterile forceps, placed on a sterile glass slab, and lightly compressed with sterile gauze to obtain a uniform fibrin membrane suitable for intra-canal placement. The gel obtained from the middle layer was trimmed to the canal diameter and carefully positioned directly over the transplanted pulp tissue to ensure intimate contact without exerting pressure. Coronal closure with MTA and composite resin was completed in the same manner as in group 1 (Fig. 1).

### Control group (Group 3)

Root canal preparation was performed in the same manner in the control group. After irrigation, the canals were filled using gutta-percha and a canal sealer (AH Plus, Dentsply, Johnson City, TN, USA) with the lateral condensation technique, and restoration was completed using standard methods.

### Clinical and Radiographic Follow-up Assessment

The primary outcome of the study was radiographic healing of periapical lesions assessed using cone-beam computed tomography (CBCT) and standardized periapical radiographs. The secondary outcome was recovery of pulp sensibility assessed by electrical pulp testing (EPT). Clinical findings, including spontaneous pain, swelling, sinus tract formation, and percussion sensitivity, were recorded at each follow-up visit as indicators of clinical status.

All patients were followed up for a minimum of 12 months. Follow-up sessions were conducted at the 3rd, 6th, and 12th months after the intervention. Radiographic assessment was performed using both standardized periapical radiographs and cone-beam computed tomography (CBCT). CBCT was used for quantitative assessment of periapical lesion size at baseline and at the 12-month follow-up, whereas standardized periapical radiographs were used at follow-up visits to support the radiographic evaluation by confirming the presence, persistence, or reduction pattern of periapical radiolucency over time. For CBCT analysis, lesion size was measured on the axial slice showing the greatest radiolucent dimension, using a calibrated measurement tool to ensure reproducibility across timepoints. Volumetric analysis was not performed because the small sample size and irregular lesion morphology could introduce substantial segmentation variability. Therefore, a standardized linear measurement of the maximum lesion diameter was used to allow consistent and reproducible comparisons across follow-up time points, a method commonly adopted in clinical endodontic outcome studies. Pulp sensibility was assessed using an electric pulp tester (EPT) (Digitest II; Parkell Inc., Edgewood, NY, USA). Before testing, the tooth surface was isolated and gently dried. The probe tip was placed on the middle third of the buccal surface of the tooth using a conducting medium. Adjacent and contralateral teeth were also tested as controls to confirm the reliability of the patient’s response. A positive response was defined as any reproducible sensation reported by the patient before reaching the maximum output of the device. Because electric pulp testing evaluates neural response rather than true vascular pulp vitality, EPT was used in this study to detect the possible return of sensibility response during follow-up.

### Statistical analysis

Statistical analyses were performed using SPSS software (version 28.0; IBM Corp., Armonk, NY, USA). Data distribution was assessed using the Shapiro–Wilk test. Given the small sample size in each group (*n* = 7), assumptions required for parametric testing could not be reliably confirmed; therefore, non-parametric statistical methods were applied for all inferential analyses.

Continuous variables are presented as mean ± standard deviation (SD) for descriptive purposes and to facilitate comparison with previously published studies. Lesion size measurements are reported as mean ± SD in Table 1 for descriptive clarity; however, all statistical comparisons were conducted using non-parametric tests.

**Table 1:** Within-group changes in periapical lesion size at 12 months

Group	Baseline lesion size (mm) Mean ± SD	12-month lesion size (mm) Mean ± SD	Absolute reduction (mm)	*p* value†
PT group	5.38 ± 1.84	1.48 ± 0.30	3.90	0.002
PT + CGF group	5.43 ± 2.11	2.24 ± 1.25	3.19	0.002
RCT group	5.38 ± 2.59	3.13 ± 2.03	2.25	0.007

†Within-group comparisons were assessed using the Wilcoxon signed-rank test. Values are presented as mean ± SD for descriptive purposes

Intergroup comparisons were performed using the Kruskal–Wallis test, while intragroup changes over time were assessed using the Wilcoxon signed-rank test. Categorical variables were analyzed using the chi-square test or Fisher’s exact test, as appropriate. Changes in pulp sensibility status between baseline and follow-up were evaluated using McNemar’s test.

To explore the relationship between periapical lesion reduction and recovery of pulp sensibility, receiver operating characteristic (ROC) curve analysis was conducted. Given the exploratory nature of the study and the limited sample size, ROC analysis was interpreted descriptively as a hypothesis-generating analysis rather than for confirmatory inference.

All statistical tests were two-tailed, and statistical significance was set at α = 0.05.

## Results

### Radiographic healing and lesion size reduction

All three groups showed a reduction in periapical lesion size at 12 months compared with baseline. Within-group analyses indicated statistically significant changes; however, given the exploratory design and small sample size, these findings should be interpreted as descriptive and hypothesis-generating rather than confirmatory. Within-group comparisons revealed marked decreases in lesion dimensions: PT group showed a reduction from 5.38 ± 1.84 mm to 1.48 ± 0.30 mm (*p* = 0.002); PT + CGF group decreased from 5.43 ± 2.11 mm to 2.24 ± 1.25 mm (*p* = 0.002); and RCT group decreased from 5.38 ± 2.59 mm to 3.13 ± 2.03 mm (*p* = 0.007) (Table 1). Given the small sample size and distributional limitations, these intragroup changes were analyzed using non-parametric tests (Wilcoxon), and the results should be interpreted cautiously as exploratory and hypothesis-generating rather than confirmatory.

**Fig. 2:**
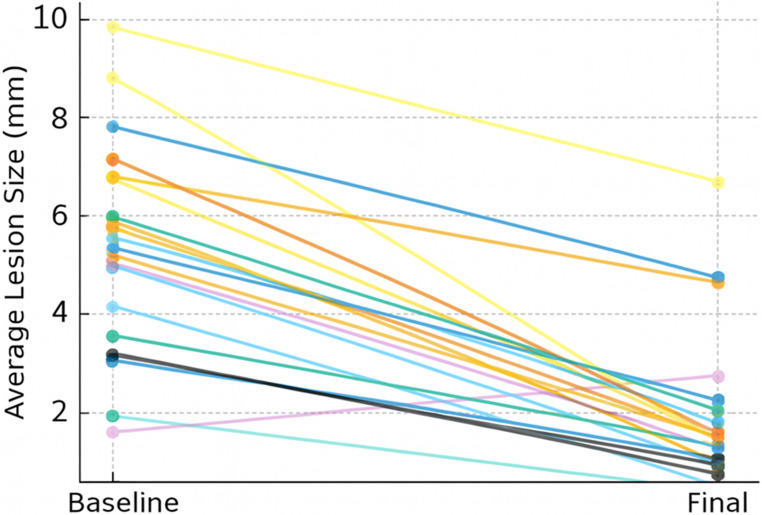
Each line represents the patient. A significant decrease was observed in all groups from the beginning to the end of the study

Intergroup comparisons demonstrated no significant differences in absolute lesion size at 12 months Non-parametric analysis confirmed that the groups did not differ significantly at 12 months (Kruskal–Wallis *p* > 0.05). Similarly, the rates of percentage shrinkage did not significantly differ among the groups (Kruskal–Wallis χ²(2) = 1.45, *p* = 0.37) (Fig. 2). Representative CBCT images illustrating baseline and 12-month periapical healing patterns across the three treatment groups are shown in Fig. 5.

### Sensibility outcomes

**Fig. 3:**
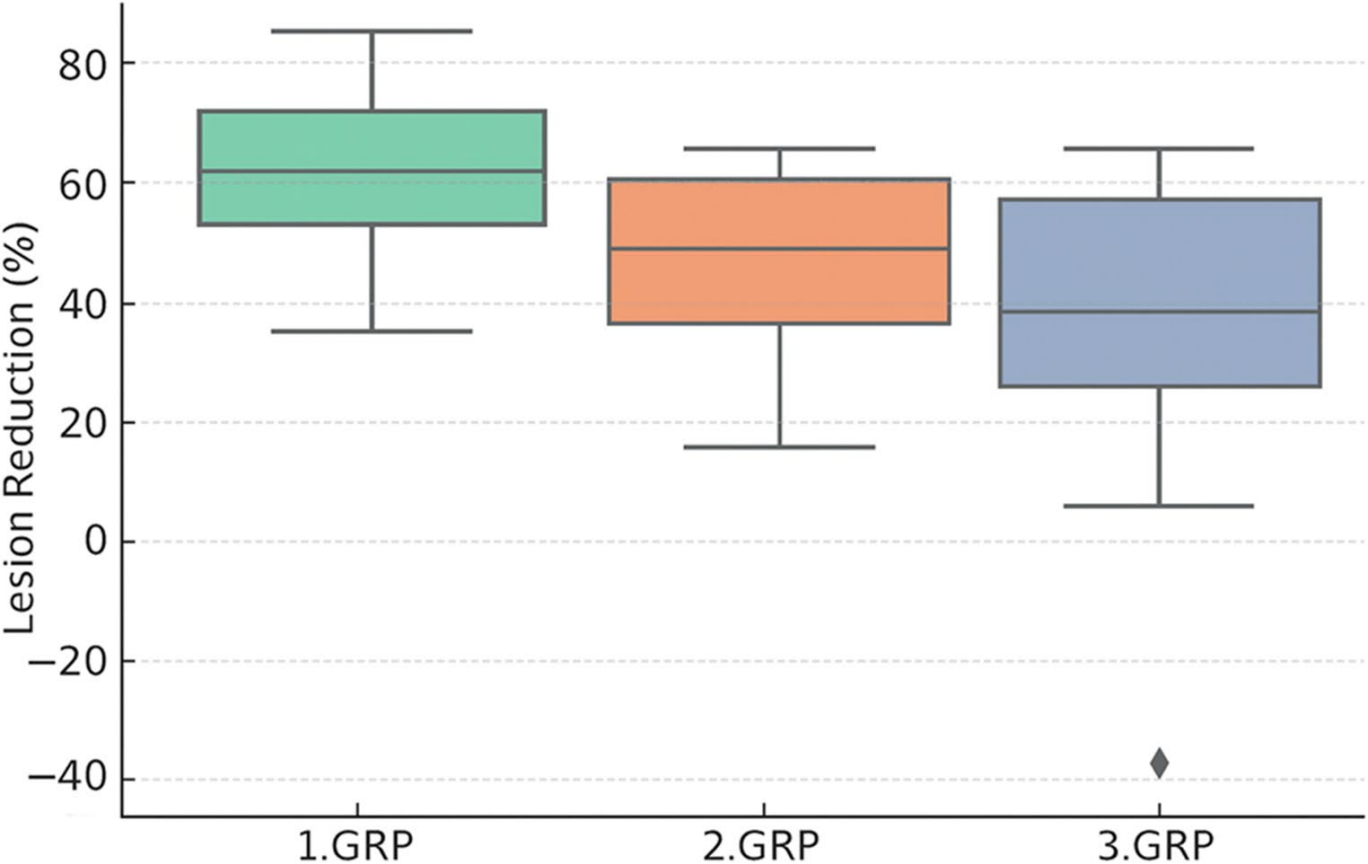
Box plot showing the median, interquartile range (IQR), and 95% confidence intervals

Among the 21 treated teeth, a positive sensibility response to EPT was observed in eight instances (38.1%). The PT group exhibited the highest rate of recovery, with five out of seven teeth (71.4%) responding positively to electric pulp testing at the 12-month mark. In the PT + CGF group, sensibility was observed in three out of seven teeth (42.9%), whereas the RCT group showed no sensibility response (0/7) (Table 2). When all teeth were considered together, the overall change in sensibility status between baseline and the 12-month follow-up was statistically significant (McNemar *p* = 0.013). This change was driven by sensibility responses observed in the pulp transplantation groups, whereas no sensibility response was detected in the conventional RCT group. Differences among the groups were not statistically significant (Chi-square χ²(2) = 1.22, *p* = 0.22) (Fig. 3). However, given the exploratory design and limited sample size, these findings should be interpreted cautiously and considered hypothesis-generating rather than confirmatory.

**Table 2:** Sensibility changes

Group	*n*	Positive response (*n*, %)	Negative response (*n*, %)
PT group	7	5 (71.4%)	2 (28.6%)
PT + CGF group	7	3 (42.9%)	4 (57.1%)
RCT group	7	0 (0%)	7 (100%)

*McNemar test result: *p* = 0.013. Overall χ²(2) = 1.22, *p* = 0.22

In Table 2, changes in sensibility are presented by group, and statistical differences are evaluated

### Predictive analysis

**Fig. 4:**
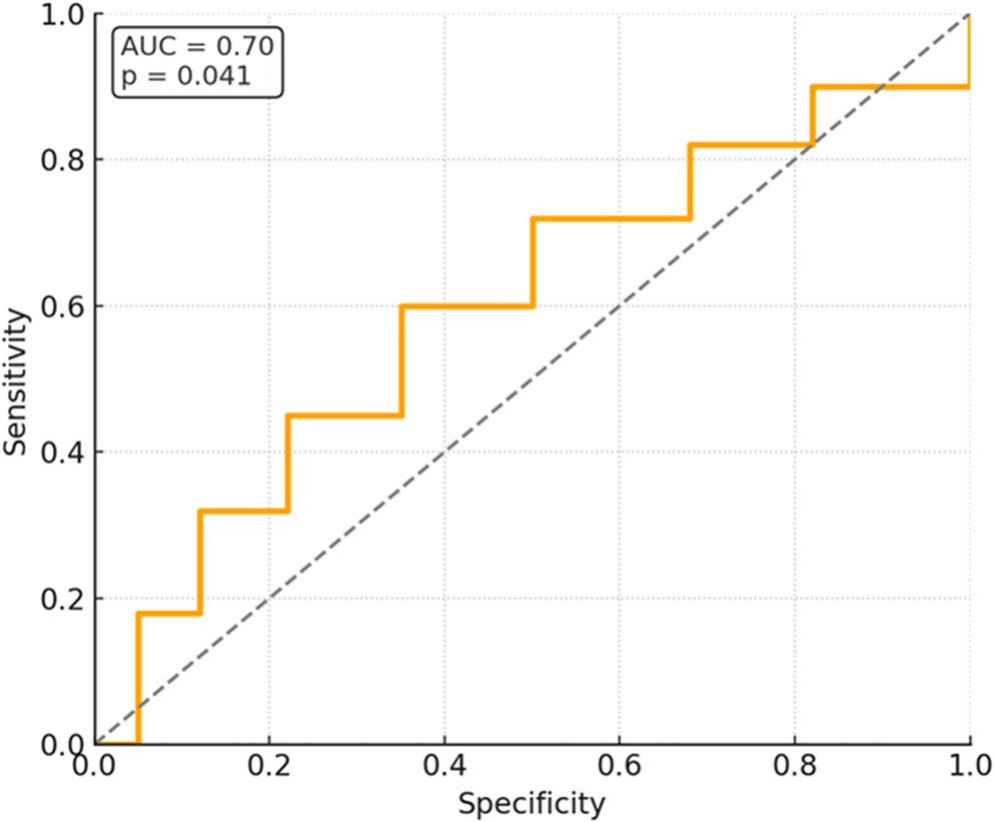
Sensitivity (Y-axis) and 1-Specificity (X-axis) are labeled in English. The orange step curve represents the classifier performance, and the dashed line is the no-discrimination line. Receiver operating characteristic (ROC) analysis suggested a possible association between periapical lesion shrinkage and sensibility response at 12 months, with an AUC of 0.70 (95% CI: 0.52–0.88; *p* = 0.041)

Receiver Operating Characteristic (ROC) curve analysis suggested a possible association between the percentage reduction in lesion size and sensibility response, with an Area Under the Curve (AUC) of 0.70 (95% CI: 0.52–0.88, *p* = 0.041) (Fig. 4). Given the exploratory design and limited sample size, this analysis should be interpreted cautiously and considered hypothesis-generating rather than predictive. Effect size calculations indicated a substantial within-group effect for the reduction in 3D lesion volume (Cohen’s d = 1.20) and a small-to-moderate intergroup effect (η²=0.07).

### Adverse events

**Fig. 5:**
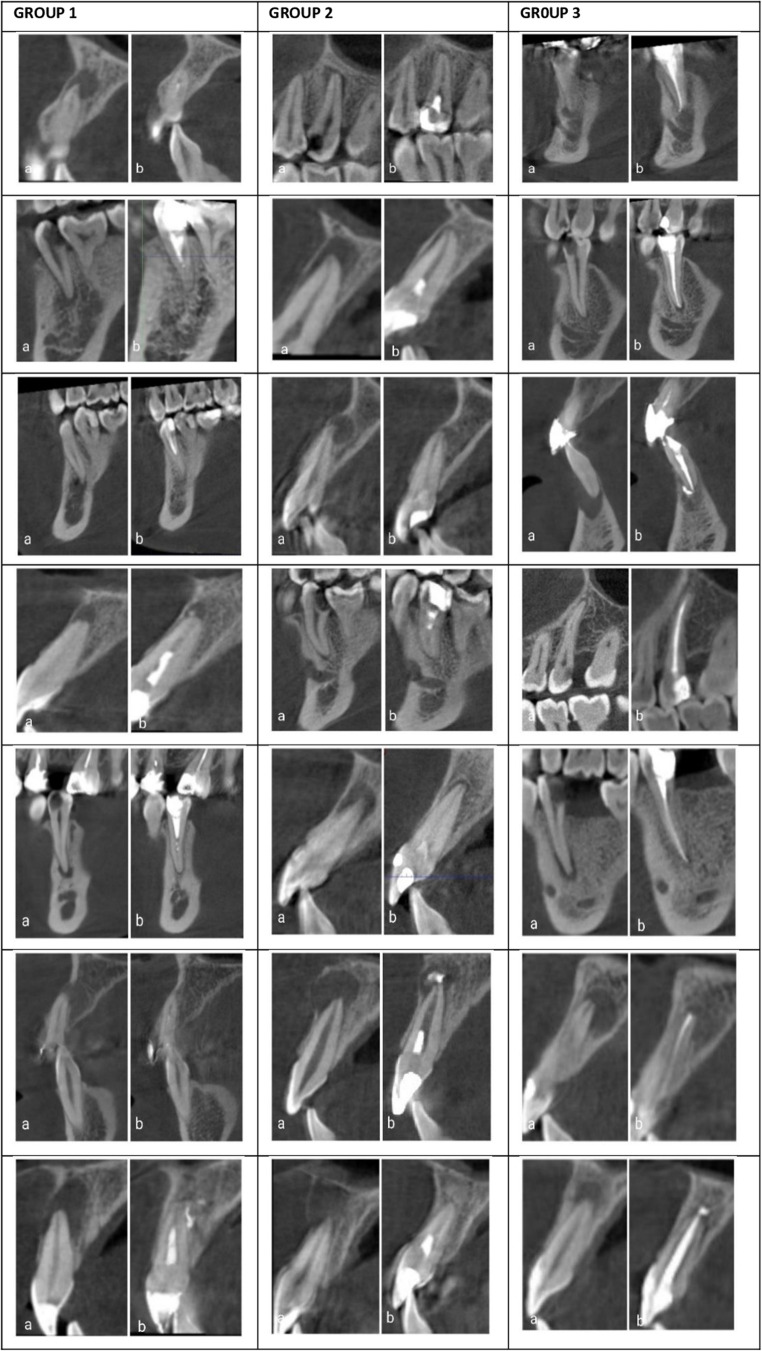
The figure is arranged by treatment groups in rows: Row 1: PT group (pulp transplantation), Row 2: PT + CGF group (pulp transplantation + concentrated growth factor), Row 3: RCT group (conventional root canal therapy). For each group, panel a show the initial radiolucency, and panel b shows the corresponding 12-month CBCT image of the same patient. All groups demonstrated lesion reduction, with the most pronounced healing observed in PT group

During the 12-month follow-up period, no adverse events, complications, or unexpected reactions were reported in any group. The procedures were well tolerated by all patients, with no instances of postoperative pain, swelling, or infection beyond what is typically expected in the clinical course (Fig. 5).

## Discussion

This study provides one of the earliest clinical indications that dental pulp transplantation may restore sensibility and promote periapical healing in necrotic mature teeth—an outcome traditionally regarded as biologically improbable. The consistent sensibility recovery and notable lesion resolution observed within 12 months suggest that mature teeth retain a degree of regenerative responsiveness previously underestimated in clinical practice. These findings challenge the prevailing assumption that regeneration in mature necrotic teeth is inherently limited and highlight the need for treatment protocols specifically adapted to the biological and anatomical constraints of adult dentition, including reduced stem cell availability, narrow apical anatomy, and a less permissive microenvironment [5, 24].

Pulp stem cells are considered the most advantageous source of stem cells due to several factors. These include their relatively straightforward isolation from the body compared to other stem cell sources, their high proliferative capacity, and the absence of transplant rejection, as all cells possess identical DNA and RNA. Furthermore, they contain fully mature connective tissue, an established neuronal network, and pre-existing vascularization [9]. As documented in the literature, pulp tissue cells harvested from the pulp of deciduous teeth [3, 10] and third molars [9, 25] have been utilized in numerous studies. Çehreli et al. [3] documented successful outcomes in the transplantation of primary tooth pulp into immature teeth. Nevertheless, the implementation of this method is limited by the frequent lack of available donor primary teeth in mature dentition.In our study, the use of third molar pulp successfully addressed the limitation of unavailable donor primary teeth in adults. Previous studies often relied on cell culture and expansion, which introduced procedural delays and increased infection risk [12, 25]. In contrast, transplanting the entire pulp tissue without culture provides clinical convenience and reduces contamination risk.

Our approach enables transplantation of the entire dental pulp without the need for cell expansion, providing both clinical convenience and cost-effectiveness. The survival of DPSCs is also enhanced when the tissue is rapidly transferred into a natural biological environment, reducing the risks of infection and hypoxia. These advantages markedly increase the clinical applicability and success of pulp transplantation.

In our investigation, apical bleeding was intentionally induced to improve the nutritional support of DPSCs; however, apical lesions often limited the degree of bleeding. Although favorable sensibility responses and periapical healing were observed in the present study, these clinical findings should not be interpreted as definitive evidence regarding the biological necessity or non-necessity of apical bleeding. Rather, apical bleeding may have acted as a supportive factor, and its precise contribution to pulp tissue survival and regeneration remains to be clarified in larger studies with histological validation.

This finding aligns with previous reports suggesting that regenerative potential may rely more on transplanted pulp tissue and dentin-derived growth factors than on a blood clot [3, 9]. In a recent study examining teeth for which apical bleeding was intended but could not be achieved, and where a blood transfusion from the extraction socket was performed during transplantation, sensibility was observed in only one out of six cases [11].

Other cell-based experiments have incorporated additional bioactive factors and scaffolding materials, including G-CSF, platelet-poor plasma, leukocyte- and PRF, and pre-clotted PRP, to support tissue regeneration [8, 12]. In our study, no growth factors were introduced to any tooth, except in Group 2, where CGF was tested. The transplantation environment relied solely on endogenous dentin-derived factors released via EDTA irrigation or minimal apical bleeding [11].

In Group 2, where pulp transplantation was conducted with the incorporation of CGF, the sensibility response and resolution of periapical lesions were less pronounced compared to Group 1, which underwent transplantation without supplementary interventions. One study found that teeth treated with CGF did not exhibit significant differences compared to other endodontic regenerative procedures [26]. In a study on regenerative treatment performed on mature teeth without pulp transplantation, the CGF and PRF groups were compared; at the 6th month, the CGF group showed a better healing response in terms of reduction in periapical lesion size compared to the PRF group. However, this change diminished considerably at the 12th month [20]. In the present study, the CGF group showed numerically lower sensibility responses and less lesion reduction at 12 months compared with the pulp transplantation group; however, these differences should be interpreted cautiously because the study was not powered to detect intergroup differences. The comparatively less favorable outcomes observed in the CGF group should be interpreted cautiously. Although one possible explanation may involve differences in the local biological environment associated with CGF application, the present study was not designed to investigate underlying mechanisms. Therefore, these observations should be regarded as hypothesis-generating only, and further experimental and histological studies are required to clarify the biological effects of CGF in pulp transplantation. However, this hypothesis requires further histological confirmation and represents a limitation of the present study.

The American Association of Endodontists recommends the use of a 1.5% sodium hypochlorite (NaOCl) solution for root canal disinfection in regenerative endodontic procedures [27]. Elevated concentrations (5.25–6%) effectively disrupt biofilms but are cytotoxic to apical stem cells and degrade dentin-derived growth factors [28–30]. These harmful effects can be largely prevented by using 1.5% NaOCl, followed by 17% EDTA [31]. EDTA also promotes the release of VEGF and TGFβ-1, which contribute to angiogenesis and stem cell activity [32, 33]. The AAE Clinical Considerations recommend low-concentration NaOCl, based mainly on in vitro evidence showing its cytotoxicity to apical papilla stem cells rather than on its antibacterial effectiveness in vivo [34]. Although the AAE recommendations apply to immature teeth, no guidelines exist for regenerative procedures in mature teeth. In our study, a 2.5% NaOCl solution was selected to balance effective disinfection with preservation of dentin-derived growth factors, given the absence of an apical papilla and the need to eliminate persistent biofilm. Based on our outcomes, this concentration appeared optimal for disinfecting the canal system without compromising periapical stem cell viability. Additionally, 17% EDTA was used to mitigate the negative effects of NaOCl and maximize the release of growth factors. In addition to NaOCl, a triple-antibiotic paste composed of Cefuroxime, Ciprofloxacin, and Metronidazole was used, avoiding minocycline-related discoloration and ensuring effective microbial control throughout follow-up.

In the broader endodontic literature, necrotic teeth with open apices are commonly managed using apexification or apical barrier techniques rather than transplantation-based regenerative approaches. Calcium silicate–based materials such as mineral trioxide aggregate (MTA) and OrthoMTA have been widely used to create artificial apical barriers in immature teeth with necrotic pulp and apical periodontitis, demonstrating favorable clinical and radiographic outcomes [35]. Long-term studies have also reported high success rates for the MTA apical plug technique in teeth with necrotic pulp and immature apices [36]. In addition, a split-mouth clinical study suggested that the apical extent of the MTA barrier may not significantly influence treatment outcomes in non-vital immature teeth [37]. However, these studies mainly involve immature teeth with open apices and therefore differ biologically from the mature necrotic teeth included in the present study.

Regarding apical enlargement, prior studies reported no improvement in sensitivity when the apical diameter was enlarged to 0.35–0.40 mm [11]. Another study presented a case in which cultured and transplanted DPSCs led to regained pulp sensitivity; the apical foramen diameter in that case was 0.35 mm [14]. In contrast, the cases included in this study maintained an apical width of 0.25–0.30 mm while still achieving positive sensibility responses. This finding differs from previous studies suggesting that larger apical diameters may favor regenerative outcomes by facilitating cell migration and vascular ingrowth, and may indicate that a narrower apical opening protects transplanted tissue from residual infection [38, 39]. Further clinical trials are required to determine the optimal apical diameter for pulp transplantation in mature teeth.

In particular, the absence of histological evaluation prevents confirmation of whether the observed sensibility responses reflect true pulp-dentin complex regeneration or another form of reparative tissue response.

Finally, the limited sample size and the nature of this study restrict the generalizability of the findings. Larger, multi-centered, and histologically validated studies are needed to confirm the biological mechanisms and clinical predictability of pulp transplantation in mature necrotic teeth.

Because of the exploratory design and limited sample size, the statistical findings of the present study should be interpreted as hypothesis-generating and should not be considered definitive evidence of treatment superiority.

## Conclusion

This study represents one of the few investigations that have implemented pulp transplantation in mature teeth with periapical lesions, and it reports the largest case series documented in the literature. Our findings suggest that pulp transplantation is a viable and promising regenerative treatment option that can be executed clinically without the necessity for laboratory conditions or specialized equipment. However, the incorporation of CGF did not appear to confer any additional benefit to the pulp transplantation. Further comprehensive and histologically supported studies are required to elucidate the effects of CGF on dental pulp stem cells.

Within the limitations of this exploratory randomized clinical trial study, pulp transplantation demonstrated the potential to promote sensibility response and periapical healing in necrotic mature teeth; however, definitive claims regarding true pulp–dentin complex regeneration cannot be made without histological verification.

Overall, these findings should be interpreted with caution due to the small sample size and exploratory nature of this clinical investigation. Larger, well-controlled studies are necessary to confirm the predictability, biological mechanisms, and long-term outcomes of pulp transplantation in mature teeth.

## Data Availability

Data are available from the corresponding author upon reasonable request. The full study protocol and statistical analysis plan are available from the corresponding author upon reasonable request. De-identified individual participant data, including the data dictionary and statistical code used for analysis, may also be obtained from the corresponding author.

## Declarations

### AI declaration

Generative AI (ChatGPT, OpenAI) was used to refine wording, enhance readability, assist in summarizing long sections, and provide structural suggestions. No AI tool was used to generate scientific data, interpret results, or create original content. The author(s) reviewed and edited all text and take full responsibility for the final version.

## References

[1] Murray PE, Garcia-Godoy F, Hargreaves KM (2007) Regenerative endodontics: a review of current status and a call for action. J Endod 33(4):377–390. 10.1016/j.joen.2006.09.013 17368324 10.1016/j.joen.2006.09.013

[2] Nakashima M, Iohara K, Bottino MC, Fouad AF, Nör JE, Huang GTJ (2019) Animal models for stem cell-based pulp regeneration: foundation for human clinical applications. Tissue Eng Part B Rev 25(2):100–113. 10.1089/ten.TEB.2018.0194 30284967 10.1089/ten.teb.2018.0194 PMC6486672

[3] Cehreli ZC, Unverdi GE, Ballikaya E (2022) Deciduous tooth pulp autotransplantation for the regenerative endodontic treatment of permanent teeth with pulp necrosis: a case series. J Endod 48(5):669–674. 10.1016/j.joen.2022.01.015 35114270 10.1016/j.joen.2022.01.015

[4] Glynis A et al (2021) Regenerative endodontic procedures for the treatment of necrotic mature teeth with apical periodontitis: a systematic review and meta-analysis of randomized controlled trials. J Endod 47(6):873–882 33811981 10.1016/j.joen.2021.03.015

[5] Liu K et al (2024) An innovative cell-based transplantation therapy for an immature permanent tooth in an adult: a case report. BMC Oral Health 24(1):646. 10.1186/s12903-024-04410-7 38824565 10.1186/s12903-024-04410-7 PMC11143573

[6] Yan H et al (2023) Regenerative endodontics by cell homing: a review of recent clinical trials. J Endod 49(1):4–17. 10.1016/j.joen.2022.09.008 36270575 10.1016/j.joen.2022.09.008

[7] Sui B et al (2019) Pulp stem cell-mediated functional pulp regeneration. J Dent Res 98(1):27–35. 10.1177/0022034518808754 30372659 10.1177/0022034518808754

[8] Kim SG (2021) A cell-based approach to dental pulp regeneration using mesenchymal stem cells: a scoping review. Int J Mol Sci 22(9):4357. 10.3390/ijms22094357 33921924 10.3390/ijms22094357 PMC8122243

[9] Feitosa VP et al (2021) Dental pulp autotransplantation: a new modality of endodontic regenerative therapy—follow-up of 3 clinical cases. J Endod 47(9):1402–1408 34175322 10.1016/j.joen.2021.06.014

[10] Tang X et al (2024) Deciduous pulp tissue implantation into the root canal of mandibular incisor resulted in pulp revascularization: a case report with a 5-year follow-up. J Clin Pediatr Dent 48(3):171–176 38755996 10.22514/jocpd.2024.071

[11] Kim U et al (2025) Regenerative endodontic procedures with minced pulp tissue graft in mature permanent teeth: a clinical study. J Endod 51(1):43–53e2 39401572 10.1016/j.joen.2024.10.004

[12] Nakashima M, Iohara K, Murakami M et al (2017) Pulp regeneration by transplantation of dental pulp stem cells in pulpitis: a pilot clinical study. Stem Cell Res Ther 8(1):61. 10.1186/s13287-017-0506-5 28279187 10.1186/s13287-017-0506-5 PMC5345141

[13] Khayat A et al (2017) GelMA-encapsulated hDPSCs and HUVECs for dental pulp regeneration. J Dent Res 96(2):192–199. 10.1177/0022034516682005 28106508 10.1177/0022034516682005 PMC5331619

[14] Meza G et al (2019) Personalized cell therapy for pulpitis using autologous dental pulp stem cells and leukocyte platelet-rich fibrin: a case report. J Endod 45(2):144–149 30711169 10.1016/j.joen.2018.11.009

[15] Liu H, Lu J, Jiang Q, Haapasalo M, Qian J, Tay FR, Shen Y (2022) Biomaterial scaffolds for clinical procedures in endodontic regeneration. Bioact Mater 12:257–277. 10.1016/j.bioactmat.2021.10.008 35310382 10.1016/j.bioactmat.2021.10.008 PMC8897058

[16] Li Z et al (2021) The effects and potential applications of concentrated growth factor in dentin–pulp complex regeneration. Stem Cell Res Ther 12(1):357. 10.1186/s13287-021-02446-y 34147130 10.1186/s13287-021-02446-y PMC8214771

[17] Xie Y et al (2023) Pulp regeneration by transplantation of dental pulp with the synergy of concentrated growth factor: an in vitro and in vivo study 10.21203/rs.3.rs-2663591/v1

[18] Wang M et al (2025) Investigation of an injectable concentrated growth factor gel for pulp tissue regeneration. Aust Endod J 10.1111/aej.70015 10.1111/aej.70015 40814847

[19] Cohen J (1992) A power primer. Psychol Bull 112(1):155–159 19565683 10.1037//0033-2909.112.1.155

[20] SSalah T, Hussein W, Abdelkafy H (2025) Regenerative potential of concentrated growth factor compared to platelet-rich fibrin in treatment of necrotic mature teeth: a randomized clinical trial. BDJ Open 11(1):10 39900647 10.1038/s41405-024-00288-3 PMC11790909

[21] Nageh M, Ahmed GM, El-Baz AA (2018) Assessment of regaining pulp sensibility in mature necrotic teeth using a modified revascularization technique with platelet-rich fibrin: a clinical study. J Endod 44(10):1526–1533 30174103 10.1016/j.joen.2018.06.014

[22] Torabinejad M, Parirokh M (2010) Mineral trioxide aggregate: a comprehensive literature review—part II: leakage and biocompatibility investigations. J Endod 36(2):190–202 20113774 10.1016/j.joen.2009.09.010

[23] Yu S et al (2023) Mechanism of pulp regeneration based on concentrated growth factors regulating cell differentiation. Bioeng (Basel) 10(5):513 10.3390/bioengineering10050513 PMC10215240 37237583

[24] Garrido-Parada S et al (2022) Endodontic regenerative procedures in necrotic adult teeth. Appl Sci 12(9):4212

[25] Nakashima M, Tanaka H (2024) Pulp regenerative therapy using autologous dental pulp stem cells in a mature tooth with apical periodontitis: a case report. J Endod 50(2):189–195 37923123 10.1016/j.joen.2023.10.015

[26] Almutairi M, Almotairy N, Alotaibi B (2025) Efficacy of concentrated growth factor compared with other types of regenerative endodontic procedures: a systematic review. BMC Oral Health 25:974 40597984 10.1186/s12903-025-06358-8 PMC12220363

[27] American Association of Endodontists (2016) AAE clinical considerations for a regenerative procedure. American Association of Endodontists, Chicago

[28] Jung C, Kim S, Sun T, Cho YB, Song M (2019) Pulp-dentin regeneration: current approaches and challenges. J Tissue Eng 10:2041731418819263 30728935 10.1177/2041731418819263 PMC6351713

[29] Cai C et al (2023) Advances in the role of sodium hypochlorite irrigant in chemical preparation of root canal treatment. Biomed Res Int 2023:36685672 10.1155/2023/8858283 PMC9859704

[30] Martin DE, De Almeida JFA, Henry MA, Khaing ZZ, Schmidt CE, Teixeira FB, Diogenes A (2014) Concentration-dependent effect of sodium hypochlorite on stem cells of apical papilla survival and differentiation. J Endod 40(1):51–55 24331991 10.1016/j.joen.2013.07.026

[31] Galler KM et al (2011) Dentin conditioning codetermines cell fate in regenerative endodontics. J Endod 37(11):1536–1541 22000458 10.1016/j.joen.2011.08.027

[32] Ivica A et al (2020) Transforming growth factor beta 1 distribution and content in the root dentin of young mature and immature human premolars. J Endod 46(5):641–647 32139264 10.1016/j.joen.2020.01.016

[33] Mohammadi Z, Shalavi S, Jafarzadeh H (2013) Ethylenediaminetetraacetic acid in endodontics. Eur J Dent 7(Suppl 1):S135–S142 24966721 10.4103/1305-7456.119091 PMC4054072

[34] Wei X, Yang M, Yue L et al (2022) Expert consensus on regenerative endodontic procedures. Int J Oral Sci 14(1):55 36450715 10.1038/s41368-022-00206-z PMC9712432

[35] Parolia A, Yu A, Feghali M (2022) Management of teeth with open apex and apical periodontitis using MTA and OrthoMTA: a case series and review. G Ital Endod 36(1) 10.32067/GIE.2021.35.02.43

[36] Pace R et al (2014) Mineral trioxide aggregate as apical plug in teeth with necrotic pulp and immature apices: a 10-year case series. J Endod 40(8):1250–1254 25069943 10.1016/j.joen.2013.12.007

[37] Tabiyar K, Logani A (2021) The apical extent of mineral trioxide aggregate apical barrier does not influence the treatment outcome in a nonvital immature permanent anterior tooth: a split-mouth clinical study. Eur Endod J 6(1):44–49 33609017 10.14744/eej.2020.08760 PMC8056805

[38] Fang Y et al (2018) Influence of apical diameter on the outcome of regenerative endodontic treatment in teeth with pulp necrosis: a review. J Endod 44(3):414–431 29273495 10.1016/j.joen.2017.10.007

[39] Laureys WG et al (2013) The critical apical diameter to obtain regeneration of the pulp tissue after tooth transplantation, replantation, or regenerative endodontic treatment. J Endod 39(6):759–763 23683275 10.1016/j.joen.2013.02.004

